# Miscellanea

**Published:** 1855-12

**Authors:** 


### MISCELLANEA.

### STEEL BISCUITS.

Mr. F. Allarton has forwarded for our inspection a box of his steel biscuits. After having tasted, tested, and applied them in treatment, we are bound to say that they form a very elegant and effectual mode of administering iron in small and long-continued doses ; the best way, in short, in which iron can be given. To simplify medicines, to make them palatable, and to render them as much like foods as possible, includes full half the success attendant on the treatment of diseases.

### RAILWAY TRAVELLING, AND ITS EFFECTS ON HEALTH.

BY J. M. WINN, M.R.C.P., ETC.

The practice of rushing to and from a distant watering-place daily by rail—to live at London and Brighton as it were at the same time—has not proved so conducive to health and happiness as the originators of the custom expected. It is a well known fact, that many merchants begin to repent of having planted their domestic circles far away from their counting-houses. The wear and tear consequent on this practice is very great; but the causes of the evils are not to be found in the noise, vibration, and speed of the railway carriage—although I have known these forces act prejudicially on a weak organisation—but in the excitement, anxiety, and nervous shock consequent on the frequent efforts to catch the *last* express ; to be in time for the fearfully punctual train. The jaded and anxious merchant, or professional man, feels that to miss the train by only half a minute is tantamount to losing that happiness which had supported him, and to which he had been perhaps looking forward during a day of toil, the hope of spending the evening with his family. Use, strong nerves, and a cool temperament, moderate the effects of this incessant anxiety; nevertheless, there are many who must, and do, suffer from the reiterated shocks attendant on the practice to which I refer. On the diseased, the above causes would be expected to exert a still more serious effect. Cases of sudden death, produced by the hurry and eagerness often required to reach the train in time, are on record. A valued friend of the writer's, suffering from an affection of the heart, fell down dead on the platform of a railway station; his life being the forfeit paid for the exertion which he had made to reach the starting train.

Although I admit that *incessant* railway travelling will produce directly, from the vibration of the carriage, a certain amount of cerebral irritation in highly nervous temperaments, I am quite convinced that, to the healthily organised individual (if we except the *indirect* influences referred to in the beginning of this paper), there is no more injury to be anticipated from a railway than an ordinary carriage. The difference of speed matters not. In a close, secure, and well poised vehicle, it is indifferent to the traveller's health or comfort whether he proceed at the rate of ten or of one hundred miles an hour.

I recently inquired of my friend Mr. Childs, surgeon to the Great Northern Railway, whether he had ever known an instance where an engine driver had appeared to suffer from an effect attributable to his peculiar mode of locomotion, and he assured me that he never knew of any such case. He at the same time stated that he never selected a man for the office of engine-driver, who was not in perfect health.

It is the long interval which generally elapses between the starting of each train that makes railway travelling so objectionable. Did the trains run more frequently, the nervous shocks attendant on the frequent disappointments which are experienced by those who miss the trains would be obviated. It may be said, that such disappointments are trivial. When seldom occurring they are not worthy of a thought; but if an individual be frequently subjected to them, an accumulation of such incessant petty annoyance would make a large aggregate in the course of the year.

The railway has proved a boon of incalculable value to the poor; but to the London man of business its advantages are not so obvious. To one of the latter class, who requires a country residence, more enjoyment, less anxiety, and less fatigue will be ensured if he live at a short distance from town, easily accessible by horse or carriage, than if he daily rush per rail to the Land's End and back with the speed of lightning.

Finsbury Square, August 15, 1855.

---

### STATISTICS OF BIRTHS AND DEATHS IN ST. PETERSBURGH AND MOSCOW.

*St. Petersburgh.*—The population at the end of 1852 was 532,241. The number of births during the year was 8,389 males, and 7,905 females; in all, 16,294. Of these, 7,297 males and 6,914 females, in all 14,211, were of the Russian Greek Church; and of these the legitimate births were 9,994, the illegitimate, 4,208, while 9 children were deserted by their parents.

The number of deaths in 1852 in St. Petersburgh was 21,451—13,461 males and 7,990 females, giving an excess of deaths over births equal to 5,157. The deaths of persons belonging to the Russian Greek Church amounted to 18,236: 11,593 males and 6,643 females. The number of these who died at the various periods of life are:—

| | Males. | Females. | Total. |
|---|---|---|---|
| Under 5 years | 3361 | 3072 | 6433 |
| From 5 to 10 years | 215 | 199 | 414 |
| „ 10 „ 15 „ | 482 | 146 | 628 |
| „ 15 „ 20 „ | 1024 | 265 | 1289 |
| „ 20 „ 30 „ | 2213 | 695 | 2908 |
| „ 30 „ 40 „ | 1637 | 591 | 2228 |
| „ 40 „ 50 „ | 1165 | 519 | 1684 |
| „ 50 „ 60 „ | 799 | 423 | 1222 |
| „ 60 „ 70 „ | 416 | 379 | 795 |
| „ 70 „ 80 „ | 219 | 246 | 465 |
| „ 80 „ 90 „ | 54 | 90 | 144 |
| „ 90 „ 100 „ | 4 | 14 | 18 |
| Ages unknown | 4 | 4 | 8 |
| | 11,593 | 6643 | 18,236 |

*Moscow.*—This city contained, in 1852, 375,000 inhabitants. The number of births was 9,548: 5,175 males and 4,373 females. The deaths amounted to 11,323: viz., 5,946 males and 5,377 females, being an excess of 1,775 over the births. Of these, 10,901, or 5,686 males and 5,215 females, belonged to the Russian Greek Church: the number dying at various periods of life being as under:—

| | Males. | Females. | Total. |
|---|---|---|---|
| Under 5 years | 2050 | 1932 | 3982 |
| From 5 to 10 years | 145 | 122 | 267 |
| „ 10 „ 15 „ | 164 | 93 | 257 |
| „ 15 „ 20 „ | 374 | 180 | 554 |
| „ 20 „ 30 „ | 674 | 590 | 1264 |
| „ 30 „ 40 „ | 550 | 506 | 1056 |
| „ 40 „ 50 „ | 512 | 424 | 936 |
| „ 50 „ 60 „ | 471 | 431 | 902 |
| „ 60 „ 70 „ | 398 | 430 | 828 |
| „ 70 „ 80 „ | 250 | 348 | 598 |
| „ 80 „ 90 „ | 82 | 112 | 194 |
| „ 90 „ 100 „ | 15 | 22 | 37 |
| „ 100 „ 105 „ | 0 | 1 | 1 |
| „ 105 „ 110 „ | 1 | 2 | 3 |
| „ 110 „ 115 „ | 0 | 1 | 1 |
| | 5686 | 5215 | 10,901 |

(From *Henke's Zeitschrift für die Staatsarzneikunde,* 1855.)

